# Violence suffered by Venezuelan immigrant female sex workers: an intersectional view

**DOI:** 10.1590/1980-220X-REEUSP-2023-0282en

**Published:** 2024-05-13

**Authors:** Loeste de Arruda-Barbosa, Mariana Sbeghen Menegatti, Rosa Maria Godoy Serpa da Fonseca, Maria Amélia de Campos Oliveira

**Affiliations:** 1 Universidade de São Paulo, Escola de Enfermagem, Departamento de Enfermagem em Saúde Coletiva, São Paulo, SP, Brazil.; 2 Universidade Estadual de Roraima, Faculdade de Medicina, Boa Vista, RR, Brazil.

**Keywords:** Emigrantes e Inmigrantes, Sex Work, Violence Against Women, Gender Perspective, Emigrantes e Inmigrantes, Trabajo Sexual, Violencia Contra la Mujer, Perspectiva de Género, Emigrantes e Imigrantes, Trabalho Sexual, Violência Contra a Mulher, Perspectiva de Gênero

## Abstract

**Objective:**

To characterize and analyze violence committed against Venezuelan immigrant female sex workers, from the perspective of an intersectional look at social class, gender and race-ethnicity.

**Method:**

Exploratory study with a qualitative approach. Data sources: interviews with 15 Venezuelan immigrant women sex workers and 37 Brazilian online media reports that addressed the topic. Data were submitted to thematic content analysis, with the support of Qualitative Data Analysis (WebQDA) software.

**Results:**

Thematic analysis of data from reports and interviews allowed the emergence of three empirical categories: Structural violence and reasons that led to prostitution: a question of social class; Among the forms of violence, the most feared: physical violence; Violence based on gender and race-ethnicity.

**Conclusion:**

The study made it possible to recognize that Venezuelan immigrant women who are sex workers in Brazil are subject to different types of violence and exploitation. This scenario is due to a reality of life and work that is based on the exploitation of female workers who experience the consequences of the interweaving of subalternities characteristic of their social insertion of class, gender and race-ethnicity.

## RESUMEN

**Objetivo:**

Caracterizar y analizar la violencia ejercida contra trabajadoras sexuales inmigrantes venezolanas, desde una mirada interseccional de clase social, género y raza-etnia.

**Método:**

Estudio exploratorio con enfoque cualitativo. Fuentes de datos: entrevistas con 15 trabajadoras sexuales inmigrantes venezolanas y 37 informes de medios en línea brasileños que abordaron el tema. Los datos fueron sometidos al análisis de contenido temático, con el apoyo del software Qualitative Data Analysis (WebQDA).

**Resultados:**

El análisis temático de los datos de los informes y entrevistas permitió la emergencia de tres categorías empíricas: Violencia estructural y motivos que llevaron a la prostitución: una cuestión de clase social; Entre las formas de violencia, las más temidas: la violencia física; Violencia basada en el género y la raza-etnia.

**Conclusión:**

El estudio permitió reconocer que las mujeres inmigrantes venezolanas que ejercen el trabajo sexual en Brasil están sujetas a diferentes tipos de violencia y explotación. Este escenario obedece a una realidad de vida y de trabajo que se fundamenta en la explotación de los trabajadores que experimentan las consecuencias del entrecruzamiento de subalternidades propias de su inserción social de clase, género y raza-etnia.

## RESUMO

**Objetivo:**

Caracterizar e analisar violências praticadas contra mulheres imigrantes venezuelanas profissionais do sexo, na perspectiva de um olhar interseccional de classe social, gênero e raça-etnia.

**Método:**

Estudo exploratório de abordagem qualitativa. Fontes dos dados: entrevistas com 15 mulheres imigrantes venezuelanas trabalhadoras do sexo e 37 reportagens da mídia online brasileira que abordavam o tema. Os dados foram submetidos à análise de conteúdo temática, com o apoio do software *Qualitative Data Analysis* (WebQDA).

**Resultados:**

A análise temática dos dados das reportagens e das entrevistas permitiu a emergência de três categorias empíricas: Violência estrutural e motivos que levaram à prostituição: uma questão de classe social; Entre as violências, a mais temida: a violência física; Violências baseadas no gênero e na raça-etnia.

**Conclusão:**

O estudo permitiu reconhecer que mulheres imigrantes venezuelanas profissionais do sexo no Brasil estão sujeitas a diferentes tipos de violência e exploração. Este cenário deve-se a uma realidade de vida e trabalho que se fundamenta na exploração de trabalhadoras que vivenciam as consequências do entrelaçamento das subalternizações características da sua inserção social de classe, gênero e raça-etnia.

## INTRODUCTION

The socio-economic and political crisis that has plagued Venezuela over the last decade has caused diverse phenomena, including hyperinflation, food shortages, political and social instability^(1)^. These problems have led many Venezuelans to seek better living conditions in other countries, with Brazil being one of the main destinations, especially the border state of Roraima.

Boa Vista, the capital of the state of Roraima has been impacted by the mass arrival of these immigrants on the demand for basic services such as health, education and housing, putting pressure on local resources. In spite of the shelters built to house part of this contingent of people, many live in the streets and squares of the city in a situation of extreme social vulnerability. In addition, social and economic tensions are frequent, as a result of competition for jobs and overloaded public services^(2,3,4)^.

In the field of migration studies, the phenomenon of the feminization of migration has gained prominence. In Brazil, since 2015, the spotlight has been on Haitian and Venezuelan immigrant women, most of whom are young and single, and most of whom are looking for work. However, the formal market has been unable to fully absorb this workforce^(5)^.

Unable to find formal work and living in a situation of social and economic vulnerability, some of these women end up finding prostitution as a way out to survive and to provide for their families, when they have them with them. Not infrequently, in addition to the social vulnerability resulting from their inclusion in the world of work on the bangs of the productive system, Venezuelan female sex workers are the target of prejudice and stigmatization due to their gender and nationality, thus highlighting the occurrence of xenophobia and misogyny that potentiate and intersect with other vulnerabilities, especially those of social class.

The evidence of this stigmatization can be seen in Boa Vista by the way they are pejoratively referred to, including by the media, as “las ochentas” (the eighties), a name that originates from the average price of 80 reais per sexual service. In addition to this disqualification, they are seen as intruders and targets of violence of various kinds by the local population^(6)^. It should be noted that the state of Roraima is one of the Brazilian states with the highest rates of femicide and gender violence, worsening the problem^(7,8)^.

Gender-based violence against sex workers in Roraima reflects a global challenge, as it resembles patterns observed internationally. Stigmatization, physical and sexual violence, as well as discrimination are realities shared by prostitutes in many countries, both in developed and developing nations. This problem transcends borders and occurs on all continents^(9,10)^.

As an example of this, a study that analyzed 330 cases of femicide, including prostitutes and non-prostitutes, in northwest Italy between 1970 and 2020, committed by 303 male perpetrators, showed that the majority were killed by a man they knew. The type and intensity of the relationship were the elements that most affected how the violence occurred. In intimate relationships, the risk of exaggeration, i.e. the excessive use of violence that goes beyond what is necessary to cause death, was four times higher, compared to the murder of unknown victims. In the case of prostitutes, this risk was almost four times higher for those who knew their perpetrators. The risk of being murdered with excessive violence was five times higher than for non-prostitutes. In addition, prostitutes were more likely to be victims of sexual murder, post-mortem mutilation and being killed by men with a criminal record^(11)^.

A qualitative study carried out in Guatemala in two communities of transit and destination for international or internal migrants, with 52 sex workers, examined susceptibility to violence during the stages of immigration. The results show that unsafe experiences during transit (crossing borders without documents) and negative interactions with the authorities at the place of destination (extortion, for example) contribute to increasing this population’s vulnerability to violence^(12)^.

This painful reality highlights the need for comprehensive policies and actions to tackle gender-based violence and promote the rights and safety of sex workers around the world.

Gender-based violence is a concept that encompasses different forms of violence, abuse or discrimination that occur on the basis of a person’s gender. It is a social phenomenon that impacts the lives of men and women in different ways. It is the expression of unequal power between individuals, resulting from the historical processes of constructing masculinities and femininities and the unequal power relations between men and women, women and women and men and men. This inequality materializes in the form of stereotypes and rigid gender roles that reproduce the historical subalternity of women through the use of power as domination. The social category of gender is used to understand the power relations that are established in society, differentiating biological sex from social sex^(13,14)^.

When it comes to subordination, gender violence intersects with other axes of subordination, such as social class and race-ethnicity, in an intersectional way. Intersectionality is a concept that recognizes that some social groups experience multiple forms of oppression and discrimination that intertwine and influence each other^(15)^. This means, for example, that a Venezuelan female sex worker can experience specific forms of violence that are the result of the intersection of class, gender and race-ethnicity issues and linked to the exercise of professional activity. The complexity of these forms of oppression and discrimination does not mean that one category is added to another, but that they are interrelated, both in the individual and collective expression of the social subject.

Based on the above, the object of this study is the violence practiced against Venezuelan immigrant female sex workers and its aim is to characterize and analyze the violence suffered by these women, from the perspective of an intersectional view of social class, gender and race-ethnicity.

It should be clarified that the social category generation was not considered because very little empirical data appeared that justified the use of this category of analysis or its intersectionality with the others.

## METHOD

### Type of Study

This is an exploratory study with a qualitative approach that triangulated data from two sources, namely interviews with Venezuelan immigrant women sex workers and Brazilian online media reports on the subject. The study used the Consolidated criteria for reporting qualitative research (COREQ) instrument to check all the stages of the method.

### Study Site

This study was carried out in the capital of Roraima, the city of Boa Vista.

### Selection Criteria and Sample Definition

The participants were selected according to the following inclusion criteria: being Venezuelan, over 18 years of age and a sex worker who had been in Brazil for at least six months so that they had already undergone minimal adaptation in the country. Women with dual nationality were excluded, as a nationality other than Venezuelan could interfere with the interpretation of the results when considering issues of race-ethnicity and xenophobia.

The number of participants was defined based on the data saturation technique^(16)^. According to this theoretical framework, normally 6 to 7 interviews will capture the majority of themes (80%) in a homogeneous sample. An upper limit of up to 12 interviews may be necessary to reach the highest levels of saturation.

The study used a semi-structured interview script drawn up by the authors, consisting of questions about the characteristics of the main types of actual or potential violence experienced by the participants, their context of occurrence and the interrelationships between the social categories of gender and race-ethnicity. The interviews were conducted in Portuguese or Spanish, according to the participants’ preferences. The audios were recorded, transcribed and, when in Spanish, translated into Portuguese.

Participants were recruited using the “respondent-driven sampling” method. This is a variant of chain sampling, which assumes that the components of a population that is difficult to access are more easily recruited through their peers. This method has already been used successfully in research with similar populations^(17,18)^.

### Data Collection

The interviews took place between February and March 2023. The first participant was sought out in the area where these sex workers are concentrated, in the Caimbé neighborhood, in Boa Vista, State of Roraima, Brazil, during her night shift. The interviewer was introduced, the research objectives were explained and the inclusion criteria were checked. The interviewer was a cis man, a post-doctoral researcher at the School of Nursing at the University of São Paulo, a professor of medicine with experience in qualitative research.

The participant was also informed that there would be payment for the time spent on the interview, in the amount of half the time of a client’s service, since the estimate was that the interviews would not exceed half an hour, which was confirmed. This payment was necessary because the authors understood that the interviewees were working, so there would be a financial loss if there was no payment, which could harm them.

The interviews were carried out in the interviewer’s private vehicle, in the vicinity of the interviewee’s work area. After the interview, the interviewee was asked to indicate another potential participant, who was located later and invited to take part in the research. When it was recognized that there were no new elements to elucidate the phenomenon in the material compiled during the interviews, with acknowledged repetition of information, the theoretical saturation of the data was considered and the interviews were terminated.

At the same time, documentary research was carried out by searching for reports published on online news portals such as UOL, G1, R7 and Folha de São Paulo. These sources were selected because they are recognized sites throughout the country and are among the most accessed news portals in the country^(19)^. The Folha de Boa Vista newspaper was selected to investigate local reports, due to its relevance in the state of Roraima and the notable number of reports on the subject.

The search for reports was carried out using the following expressions: “prostitutas venezuelanas”, “garotas de programa venezuelanas”, “prostituição de venezuelanas” (“Venezuelan prostitutes”, “Venezuelan call girls”, “Venezuelan prostitution”) in the search tab on the homepage of each of the portals mentioned. Next, the same expressions were entered into the google news (GN) tool on the Google search engine and reports from other local news portals were included.

We included news from online portals published from 2015, the year in which the migration of Venezuelans to Roraima intensified, until June 2022. We selected all the reports that dealt centrally with the object of study and excluded those that mentioned the fact, without exploring its triggers, or that involved women of nationalities other than Venezuelan.

### Data Analysis and Processing

The set of reports was saved as Portable Document Format and shared in a folder on the Google Drive platform for analysis. To record the data, a spreadsheet was developed containing the following information: number of the report; title; name of the media outlet; gender of the author of the report; year of publication; excerpts that addressed gender and/or race-ethnicity issues.

The data was submitted to thematic content analysis, which comprises three phases: 1) pre-analysis, in which the material was organized, followed by 2) exploration, in which the information was aggregated into symbolic or thematic categories and, finally, 3) treatment of the raw results and their interpretation, in order to then propose inferences^(20)^, with the support of the Qualitative Data Analysis software (WebQDA). Using the coding system, the software allowed nine tree codes to emerge, which were then unified into three empirical categories: Structural violence and the reasons that led to prostitution: a social class issue; Among the violence, the most feared: physical violence; Violence based on gender and race-ethnicity. The respective empirical data was analyzed in order to understand the object of study, using the analytical categories of social class, gender and race-ethnicity.

### Ethical Aspects

The study was cleared in 2022 by the Research Ethics Committee of the State University of Roraima, under opinion number 5.384.985. This study complies with Resolution 466/12. All the participants signed an informed consent form.

The term “reportage” was used to identify all the texts published on the portals accessed. The excerpts reproduced, when from a report or an interview, were identified with the letters R or E, respectively, followed by Arabic numerals to identify their sequence.

## RESULTS

Fifteen Venezuelan female sex workers were included in the study as interviewees, with ages between 18 and 46, most of them between 22 and 29 (n = 09). The majority had lived in Brazil for more than three years (n = 09). The average time spent in prostitution was three years and 6 months and none had worked or considered working as a sex worker while living in Venezuela. Most were single (n = 8), black or brown (n = 10), heterosexual (n = 14), with one or more children (n = 12). All had completed high school, one had incomplete higher education and another had completed technical education. The average monthly income from prostitution was 1,600 reais (n = 5), just over the minimum wage in Brazil at the time^(21)^. It’s worth noting that the majority (n = 10) didn’t know, didn’t want to say or were uncomfortable with the question about their monthly income and didn’t answer.

The documentary search found 37 news articles published between 2015, the year in which migration to Brazil began to intensify, and 2022. The year with the highest number of publications was 2018 (n = 15) and the year with the lowest number of publications was 2020 (n = 0). The portals with the most articles on the subject were Folha de Boa Vista and G1 (n = 8). As for the authorship of the reportages, 14 of them did not cite an author, 12 of them had male authorship, 10 had female authorship and one had shared authorship.

The analysis of the data from the reports and interviews led to the emergence of three empirical categories: Structural violence and the reasons that led to prostitution: a question of social class; Among the violence, the most feared: physical violence; Violence based on gender and race-ethnicity.

### Structural Violence and the Reasons that Led to Prostitution: A Question of Social Class

The financial crisis and the lack of job opportunities in the country of origin, combined with the reduced job opportunities on arrival in the countries of destination, Colombia and Brazil, have led many women to look to prostitution as a quick way of earning money and supporting themselves and their families. This is a consequence of the extreme poverty caused by the lack of opportunities to formally enter the productive system in the country of destination.

The testimonies quoted in the reports also highlight situations of extreme financial difficulty for the women, including a lack of money even to buy food. Many were unable to work in Brazil in the areas of academic and professional training they had obtained in Venezuela. Some were working two jobs, but even so, they were unable to meet their basic needs before opting for prostitution.

*If someone ... goes out with a client at night and arrives the next day... they’ve already earned the minimum wage in Venezuela* (R3).

*One day, I couldn’t take it anymore. My children said to me: Mom, I’m hungry. They were so hungry that their stomachs hurt. I had nothing to give them. I can cope, but they can’t* (R9).

*...shows her frustration and anxiety in a cry that she is still trying to contain. What’s the point of studying if you can’t earn money from your work? The former nursing student is now barely able to take care of her own body. She feels impure. Vile. Weak. I just want a job. A decent job* (R11).

*I haven’t known what to eat for two days, both because of the pain and the lack of money. Sometimes I think that if I had a gun I would have left by now, I can’t stand suffering like this any longer,” she vented* (R20).

The interviewed women were unanimous in reporting that the challenges to their subsistence and that of their dependents and family members were the financial need combined with the difficulty of entering the formal job market in Brazil, a situation that led them to work as sex workers. Because of the social vulnerability they experienced in Venezuela and anticipating difficulties in integrating into the formal Brazilian labor market, some had already left their country of origin to work as sex workers in Brazil. Others took up prostitution after being unable to enter the formal labor market in Brazil due to difficulties with documentation or low pay in other informal jobs.

*I started out of necessity. Cleaning for 50 reais a day is not enough. I have to pay rent, I have to pay for electricity, water, I have a son too, he wears diapers... he drinks a lot of milk. But you can’t go far with 50 reais a day here in Boa Vista* (E12).

*I went everywhere to leave my CV, I had all my documents, but I couldn’t get a job. So, I started here* (E13).

None of the women interviewed showed any satisfaction in working as a sex worker and all of them stressed that they worked as prostitutes only for financial gain. A direct relationship was identified between economic vulnerability and unprotected sex, highlighting the use of financial means as a form of social exploitation. Despite this, some of the women said that they were indignant and repulsed by men who offered them more money to have sex without a condom, showing that they were aware that this behavior could be dangerous.

*They tried to pay me 500 to do it without a condom, but I won’t* (E8).

*I think the guys must be sick to offer more money to have sex with a call girl without a condom... Then I start swearing, really swearing [laughs]* (E11).

One of the reports identified Venezuelan call girls working in Colombia, in a more vulnerable situation, who ended up giving in to unprotected sex in exchange for higher pay. Another article reported on Venezuelan prostitutes in Roraima who, at the height of the pandemic, due to the reduction in the number of clients because of social isolation, ignored the risks and served clients even when they had Covid-19.

*A recurring practice among sex workers in Cúcuta: agreeing to sex without a condom so that clients will pay more. Many know the risks, but say they accept because they need the money. They do it for survival* (R9).

*Two interviewees said that they do it even if the man is infected with the virus [Covid-19], but they charge more because they need to survive* (R20).

The economic vulnerability of these women predisposes them to other social violence, such as the exploitation of sexual services by third parties:

*Venezuelan women were sought out by nightclub owners [Roraima] who took advantage of their situation of economic vulnerability to exploit them sexually* (R29).

*The conditions in which they work as prostitutes are “inhuman”. Nine Venezuelan women living in a house where they were forced to exchange sex for food. Sixteen Venezuelan women living illegally in the country were found in a nightclub in the capital* (R29).

### Among the Violence, the Most Feared: Physical Violence

Different forms of violence were reported in the print media and by the women interviewed, such as verbal and physical violence, rape and exploitation, confirming this as a recurring and worrying problem in the region.

Among the different forms of violence suffered, physical violence was the predominant one and the one that most affected the interviewees. It was therefore the most feared.

*I was raped and almost killed. The same guy who did it to me did it to my niece too. But after three years.*” (E1).

*A boy, about 17 years old.... When I turned my back, he hung me and had a knife. I started to struggle and fell to the ground. Even my knees hurt [pause with a sigh] ... I broke free and ran...* (E2).

*I’ve been punched. When I don’t do what they want, they hit me. Almost all call girls go through this* (E3).

There were also frequent reports of theft, refusal to pay after the sexual act and verbal violence:

*Once I left my bag in the room (laughs), went to the bathroom and when I came out the guy stole my money and the guy paid with the same money he stole from me (laughs), then I started crying when I realized* (E10).

One of the reports showed how homophobia, sexism and prejudice are intrinsically linked and result in physical violence, even against women who are not sex workers:

*There was a very ugly situation I went through there in Roraima. There was a lot of prejudice there. On two occasions, when I went out on the street and met two transvestites, they hit me in the face, on my leg, on my arms because they thought I also lived on the street prostituting myself and selling my body. But that wasn’t true,” says Gabi, as she is known. “There’s this confusion that all trans girls are prostitutes, and we’re not prostitutes* (R2).

### Violence Based on Gender and Race-Ethnicity

The reports showed that, in addition to class and gender discrimination, there is discrimination based on nationality. There are reports of competition and hostility between Colombian and Venezuelan prostitutes, with Colombian prostitutes expressing dissatisfaction at the presence of Venezuelan prostitutes. This reflects the existence of tensions between different ethnic groups that can lead to marginalization and prejudice. In many cases, when referring to Venezuelan sex workers, pejorative terms are used, such as “venecas”, a derogatory term for the nationality of Venezuelan women.

*The problem that has arisen is that of the ‘venecas’ who are stealing our market because they charge less, says the Colombian prostitute* (R9).

Venezuelan women are identified as the predominant victims of rape and sexual violence in Roraima. The aggressors often take advantage of the fact that the sex workers are immigrants to commit violent acts against them. This violence is based on the feeling that because they are immigrants, indigenous or both, they are less protected.

*The highest rate of rape in Roraima has been against Venezuelan women. Most of the aggressors have taken advantage of the fact that the foreign women work as call girls to use violence* (R6).

*They offered me a job as a maid, to sleep on the premises and earn less than the minimum wage. I’m not going to earn less than the law just because I’m Venezuelan. Desperation drove me to it (prostitution). I wish Brazilians didn’t feel so much contempt for us. We’re human beings too* (R30).

*When I called the police [after being beaten], the officers saw that he was Brazilian and didn’t do anything, and the officers didn’t believe anything I said* (E2).

The contingent of Venezuelan women seeking refuge in Roraima includes a large number of black and indigenous women who end up working as sex workers. In this way, there is an accumulation of discrimination that results in greater vulnerability to violence: sex work itself, being an immigrant, being poor, being a woman and being indigenous or black.

*I feel discriminated against more for being black* (E15).

*He started hitting me, calling me black, a whore* (E1).

## DISCUSSION

This study has shown the main forms of violence suffered by Venezuelan migrant sex workers in Brazil. Most of them entered this occupation for financial reasons and are not satisfied with prostitution. These women are frequent targets of different forms of violence related to gender, social class and race-ethnicity, with physical violence being the most feared by them.

In general, the profile of the participants in this study is in line with that of other studies of Brazilian female sex workers. A 2010 survey of 2,523 sex workers in ten Brazilian cities revealed that around half of them had never been married, had little schooling and had been in the profession for less than six years^(22)^.

In another survey of 69 female sex workers, the majority were aged between 18 and 35 (78.2%), had low levels of education (53.6%), reported being black (59.4%) and had been working in the profession for less than five years (68.1%)^(23)^. Other studies have also shown that around half of the female sex workers taking part were under 30^(22,24)^.

Regarding the motivation for entering prostitution, the results of this study concur with the findings of Couto et al^(23)^, who identified that the only objective of call girls was to secure money to survive, to buy goods and to provide quality of life for themselves and their families. In the international context, it has also been found that the main reason for entering the prostitution market is for financial reasons^(10)^

All the interviewed women reported being offered additional payment for unprotected sex, which aggravates the situation of vulnerability, as the women are tempted to accept the risk of sexually transmitted infections (STIs) and other problems, in exchange for immediate financial benefit. The refusal of all the women interviewed to have unprotected sex indicates a greater awareness of the importance of preserving health, despite the adverse circumstances. It is known that satisfying male sexual desires and fantasies by paying for unsafe sex carries an increased risk of various health problems such as depression, induced abortion and STIs^(22)^.

The pattern of more vulnerable Brazilian female sex workers living in the North and Northeast regions has been identified. The percentage of women who reported using condoms in all sexual relations with a steady partner was low, ranging from 34.8 to 38.9%, suggesting a more trusting relationship with steady partners. In sexual relations with clients, the percentage of condom use was higher, ranging from 80.5 to 81.1%^(25)^.

However, every Venezuelan female sex worker in this study reported always practicing safe sex in consensual relationships with clients. In this way, they appear to be more empowered and sensitive to the risks inherent in their work and unprotected sex. It is worth considering the possibility of an information bias, as there may be a fear of judgment when assuming that the interviewer does not use condoms in relationships with clients. We must therefore consider that this data requires caution. Undoubtedly, more studies are needed to verify the veracity and relevance of this information.

Another frequent form of social violence is the sexual exploitation of female sex workers by third parties. Some reports mentioned women who were exploited by nightclub owners and owners of establishments where they practiced prostitution, even exchanging sex for food. Although prostitution is a non-criminalized occupation in Brazil, there are many violations of the human rights of these workers. The requirement to share part of their earnings with third parties is considered exploitation of sex work and is not legally permitted^(26)^.

It should be borne in mind that the capitalist system of production itself is only reproduced through the exploitation of the labor of one class over another. In the case of prostitution, it’s no different, adding gender and race-ethnicity to class exploitation. The interviewees’ speeches and the content of the reports show that the exploiters or aggressors were generally men and of Brazilian nationality.

The data in its entirety showed that these women are often subjected to sexual, physical, verbal, financial and psychological violence, reinforcing the importance of analyzing it in the light of the categories of social class, gender and race-ethnicity, in order to understand its genesis and thus be able to discuss, propose and tackle the problem.

It is understood that violence is one of the manifestations of unequal power relations, which involve the domination of rich over poor, men over women and, in the case of this study, Brazilians over Venezuelans. These relationships have a historical construction and are based on inequality between classes, genders and ethnicities, which are socially naturalized and which structurally, group and individually disadvantage poor women and women of non-white ethnicity in relation to white men^(27)^. From an international perspective, one study showed that in Bogotá, gender stigmas and violence against female sex workers are also correlated with the social machismo and ideal of masculinity present in Latin America^(9)^.

Related to gender, socially constructed stereotypes promote a double standard in relation to the exercise of sexuality. Women rent out their bodies to men for sexual pleasure. This search for pleasure is supported and reinforced as a badge of masculinity. In other words, the prostitution of women confirms a male privilege in relation to sexuality and, at the same time, disqualifies the woman who prostitutes herself. In this way, the stigmatization of prostitutes can obscure the gender inequalities that mark their social insertion. Women who prostitute themselves are seen as intrinsically bad, which in a way justifies their reduced access to rights and resources, which increases their vulnerability to various health problems, especially those linked to sexual and mental health^(22)^.

Evidence shows that Roraima is one of the most dangerous states to be a woman and has high rates of gender-related violence^(7,8)^. In addition, Venezuelan female sex workers suffer violence, oppression and violations based on race and ethnicity.

In spite of that, it is known that hierarchizing oppressions does not help us understand social issues. However, by observing what happens at the intersection of these oppressions, a broader understanding of the phenomenon studied can be achieved^(28)^. With regard to discrimination and violence against Venezuelan female sex workers, the impact of racism and stereotypes that reproduce social inequality cannot be ruled out.

Research has shown the inequality between the incomes of immigrant men and women and highlighted the differences in salaries between nationalities, with the average income of immigrant women being lower than non-immigrant men and women. It also points out that the formal labor market has not fully absorbed this workforce^(5)^.

The overlapping of stigmatizing life conditions, such as being a woman, poor, a migrant, with indigenous or black features, results in a context marked by greater vulnerability to humiliation, stigmatization and real or potential violence in their work and life context. When they are called black whores or ‘venecas’ or ‘ochentas’, it becomes clear that racial and nationality discrimination goes hand in hand with class and gender discrimination. It is therefore essential to recognize the complexity of the reality experienced by these women and to analyze it considering that its determination is due to a complex interweaving of subordinations, the product of a historical construction of inequalities that are potentiated from the structural level of objective reality, to the singularity of each woman, mediated by what happens in this specific population group.

A limiting aspect of this study may have been the value judgment of the interviewee to verbalize the answer to the question directly to a male interviewer, which can also generate fear. In an attempt to reduce this effect, it was decided to also consider data from news reports to corroborate the findings of the interviews. In any case, these findings are highly relevant to understanding the problem, as they provide a deeper insight into the lives of these women and their setbacks.

Regarding the nursing, profession, recognizing the phenomenon and its determination can serve as a motto for providing more effective care that is compatible with the needs of this specific population, especially within the scope of Primary Health Care. This type of care is the closest to the concrete reality of the population’s living and working conditions, and is therefore a privileged locus of care.

## CONCLUSION

Through the lens of the social class, gender and race-ethnicity categories, this study has made it possible to recognize that Venezuelan immigrant women sex workers in Brazil are subject to different types of violence and exploitation. This scenario is due not only to the lack of opportunities in the formal job market, but to a reality of life and work that is based on stigmatization and exploitation of women who experience the consequences of the intertwining of subalternizations characteristic of their social insertion of class, gender and race-ethnicity.

The social and economic fragility initially experienced and described by the interviewees and the reports is just the trigger for a complex situation that deepens when the reflections of unequal, historically and socially constituted power relations are recognized. Studies with an intersectional approach offer fundamental analytical possibilities for capturing and understanding the articulation of various inequalities coined in issues addressed by the categories of gender, race-ethnicity and social class.

In view of the results obtained in this study, it is suggested that further research be carried out, using different methodological strategies, to broaden and deepen our understanding of the living conditions of these women, with a view to developing public policies to overcome these vulnerabilities and protect against violence, especially in the field of public health.

## References

[1] Pinto LC, Obregon MFQ (2018). A crise dos refugiados na Venezuela e a relação com o
Brasil. Derecho y Cambio Soc.

[2] Leal NAC, Silva SM, Silva Neta ELM, Salhah S, Dalpasquale PLM, Barbosa LA (2022). Refugiados Venezuelanos em abrigos de roraima: convivência,
higiene, segurança e saúde dos abrigados. SANARE – Rev Políticas Públicas [Internet].

[3] Silva PS, Arruda-Barbosa L (2020). Imigração de venezuelanos e os desafios enfrentados por
enfermeiros da atenção primária à saúde. Enferm em Foco [Internet].

[4] Arruda-Barbosa L, Silva Neta ELM, Teixeira LDG, Silva SM, Brasil CO, Leal NAC (2020). Aspectos gerais da vida de imigrantes em abrigos para
refugiados. Rev Bras em Promoção da Saúde [Internet].

[5] Tonhati TMP, Macêdo M (2021). Os impactos da pandemia de Covid-19 para as mulheres imigrantes
no Brasil: mobilidade e mercado de trabalho. Soc e Estado [Internet].

[6] Almeida AFA, Silva LF, Lara CAS (2019). Las ochentas: o preço do refúgio. Percurso [Internet].

[7] Andrade GP, Bezerra SS (2020). Violência doméstica contra mulheres em Roraima e o uso de
tecnologias como mecanismo de enfrentamento. Rev Educ e Humanidades.

[8] Montana M (2019). Da invisibilidade da violência ao feminicídio: por que Roraima é
o estado brasileiro mais perigoso para ser mulher?. Rev Estud e Pesqui Avançadas do Terc Set.

[9] Vergara CI, Solymosi R (2022). Correlates of Client-Perpetrated Violence Against Female Sex
Workers in Bogotá. Violence Against Women.

[10] Malama K, Sagaon-Teyssier L, Parker R, Tichacek A, Sharkey T, Kilembe W, Inambao M, Price MA, Spire B, Allen S (2021). Client-Initiated Violence Against Zambian Female Sex Workers:
Prevalence and Associations With Behavior, Environment, and Sexual
History. J Interpers Violence.

[11] Zara G, Theobald D, Veggi S, Freilone F, Biondi E, Mattutino G, Gino S (2022). Violence Against Prostitutes and Non-prostitutes: An Analysis of
Frequency, Variety and Severity. J Interpers Violence.

[12] Rocha-Jiménez T, Brouwer KC, Silverman JG, Morales-Miranda S, Goldenberg SM (2016). Migration, violence, and safety among migrant sex workers: a
qualitative study in two Guatemalan communities. Culture, Health & Sexuality.

[13] Egry EY, Fonseca RMGS, Oliveira MAC (2013). Ciência, Saúde Coletiva e Enfermagem: destacando as categorias
gênero e geração na episteme da práxis. Rev Bras Enferm [Internet].

[14] Fornari LF, Fonseca RMGS (2023). Critical-emancipatory educational intervention through games to
face gender violence. Rev Bras Enferm [Internet].

[15] Kyrillos GM (2020). Uma Análise Crítica sobre os Antecedentes da
Interseccionalidade. Rev Estud Fem [Internet].

[16] Guest G, Namey E, Chen M (2020). A simple method to assess and report thematic saturation in
qualitative research. PLoS One [Internet].

[17] Lima FSS, Merchán-Hamann E, Urdaneta M, Damacena GN, Szwarcwald CL (2017). Factors associated with violence against female sex workers in
ten Brazilian cities. Cad Saude Publica [Internet].

[18] Heckathorn DD (1997). Respondent-Driven Sampling: A New Approach to the Study of Hidden
Populations. Soc Probl [Internet].

[19] Metrópoles entra para o top 3 dos sites de notícias mais lidos do
país [Internet] (2021). Metropoles.

[20] Bardin L (2011). Análise de conteúdo.

[21] Agência Câmara de Notícias (2023). Medida provisória aumenta salário mínimo para R$ 1.320 a partir
de maio [Internet]. Agência Câmara de Notícias.

[22] Villela WV, Monteiro S (2015). Gênero, estigma e saúde: reflexões a partir da prostituição, do
aborto e do HIV/aids entre mulheres. Epidemiol e Serviços Saúde [Internet].

[23] Couto PLS, Montalvão BPC, Vieira ARS, Vilela ABA, Marques SC, Gomes AMT (2020). Social representations of female sex workers about their
sexuality. Investig y Educ en Enfermería [Internet].

[24] Couto PLS, Gomes AMT, Porcino C, Rodrigues VV, Vilela ABA, Flores TS (2020). Entre dinheiro, autoestima e ato sexual: representações sociais
da satisfação sexual para trabalhadoras sexuais. Rev Eletrônica Enferm.

[25] Braga LP, Damacena GN, Szwarcwald CL, Guimarães MDC (2021). Sexual, reproductive health and health status of female sex
workers in 12 Brazilian cities, 2016. Rev Bras Epidemiol [Internet].

[26] Levate LG, Miranda RHGFC, Maciel GCCR (2020). Direito à prostituição. Dom Helder Rev Direito [Internet].

[27] Fonseca RMGS, Oliveira RNG, Gessner R (2017). PROENF-Programa de Atualização em Enfermagem: Atenção Primária e Saúde
da Família: Ciclo 5.

[28] Romano AQT, Pizzinato A (2019). Migração de mulheres para o Brasil: interseções de gênero,
raça/etnia e classe. Trab Soc.

